# Pleomorphic lobular carcinoma in a cat: Clinical, histopathological, and immunohistochemical characterization

**DOI:** 10.29374/2527-2179.bjvm001725

**Published:** 2025-05-30

**Authors:** Júlia Gabriela Wronski, Maíra Meira Nunes, Érica Almeida Viscone, Evelyn Ane Oliveira, Mariana da Silva Figueiredo, Geovanni Dantas Cassali, Karen Yumi Ribeiro Nakagaki

**Affiliations:** 1 Programa de Pós-Graduação em Patologia, Universidade Federal de Minas Gerais, Belo Horizonte, MG, Brazil.; 2 Celulavet Centro Diagnóstico Veterinário, Belo Horizonte, MG, Brazil.; 3 Veterinarian Autonomus, Belo Horizonte, MG, Brazil.; 4 Veterinarian, DSc. Universidade Federal de Minas Gerais, Belo Horizonte, Minas Gerais, Brazil.

**Keywords:** breast carcinoma, invasive lobular carcinoma, stage IV, pleural effusion, carcinoma mamário, carcinoma lobular invasivo, estágio IV, derrame pleural

## Abstract

Mammary carcinomas in cats are highly aggressive neoplasms, often with the development of metastasis. Pleomorphic lobular carcinoma, on the other hand, is an invasive carcinoma with aggressive behavior, rarely diagnosed in humans and dogs. This report describes a case of pleomorphic lobular carcinoma in cats. The histopathological and immunohistochemical features of the neoplasm in this species were characterized. The neoplasm was diagnosed in a 10-year-old cat, with regional lymph node involvement at the time of diagnosis, but without systemic signs based on imaging tests performed prior to mastectomy. However, respiratory clinical signs developed approximately 40 days after surgery, accompanied by neoplastic pleural effusion, and the patient was euthanized due to the onset of neurological signs and poor prognosis. Although uncommon, pleomorphic lobular carcinoma is a neoplasm that can affect cats, and given its poor prognosis, it is important for oncologists and pathologists to be familiar with this entity.

## Resumo

Carcinomas mamários em gatos são neoplasias altamente agressivas, frequentemente associadas ao desenvolvimento de metástases. O carcinoma lobular pleomórfico, por sua vez, é um carcinoma invasivo de comportamento agressivo, raramente diagnosticado em humanos e cães. Este relato descreve um caso de carcinoma lobular pleomórfico em um gato. As características histopatológicas e imuno-histoquímicas da neoplasia nesta espécie foram caracterizadas. A neoplasia foi diagnosticada em uma gata de 10 anos, com envolvimento de linfonodos regionais no momento do diagnóstico, mas sem sinais sistêmicos evidentes nos exames de imagem realizados antes da mastectomia. No entanto, sinais clínicos respiratórios surgiram cerca de 40 dias após a cirurgia, acompanhados de derrame pleural neoplásico, e a paciente foi eutanasiada devido ao início de sinais neurológicos e prognóstico reservado. Embora incomum, o carcinoma lobular pleomórfico é uma neoplasia que pode acometer gatos e, dado seu prognóstico desfavorável, é importante que oncologistas e patologistas estejam familiarizados com essa entidade.

## Introduction

Breast tumors are the third most common neoplasm in cats. Middle-aged to elderly females are most affected, regardless of breed. Approximately 80% of these neoplasms are malignant and exhibit aggressive biological behavior (Cassali et al., 2020; Morris, 2013; Mills et al., 2015; Petrucci et al., 2021; Souza et al., 2024). Cats with mammary tumors of histological grade III have a higher risk of death from the neoplasm, along with shorter survival and disease-free intervals compared to those diagnosed with grade I and II tumors (Cassali; Nakagaki, 2023; Souza et al., 2024). Clinically, cats with stage IV tumors and clinical signs associated with the presence of metastasis—mainly affecting the respiratory tract—have a shorter survival time (Petrucci et al., 2021).

Pleomorphic lobular carcinoma is a highly aggressive neoplasm that is rarely diagnosed in women (Al-Baimani et al., 2015; Haque et al., 2019; Sağdıç et al., 2024). In animal species, pleomorphic lobular carcinoma has only been described in female dogs (Cassali et al., 2002; D'Assis et al., 2016; Ressel et al., 2011; Salgado et al., 2012), and there are no reports of this tumor in female cats. This article aims to describe the histological and immunohistochemical characteristics of a case of pleomorphic lobular carcinoma of the mammary gland in a cat.

## Case report

A 10-year-old female cat presented with a history of multiple, bilateral mammary nodules. Due to the patient's age, it underwent unilateral mastectomy of the right mammary chain, and a second surgery was planned after recovery from the first. Tumors in this mammary chain were in the caudal thoracic, cranial abdominal, and caudal abdominal mammary glands. These tumors were clinically painless to palpation and measured approximately 1.0 cm in diameter. Imaging exams performed prior to surgery revealed no evidence of metastasis in the thoracic or abdominal organs. After the mastectomy, the tissue was sent for histopathological evaluation.

Macroscopically, three mammary glands were sent for evaluation, but only two nodules were identified. In the cranial abdominal mammary gland, a nodule measuring 2.0 x 2.0 x 1.7 cm was observed. It was firm in consistency, with an irregular surface, and presented a solid, homogeneous appearance with a white coloration upon sectioning. In the caudal abdominal mammary gland, a nodule measuring 2.0 x 1.5 x 1.0 cm was also noted. It was firm in consistency, with an irregular surface, and showed a solid, heterogeneous appearance with a light brown coloration upon sectioning. A macroscopically evident lymph node, measuring 1.0 x 0.3 cm, firm in consistency, with a light brown coloration and brownish areas upon sectioning, was also identified. The axillary lymph node was not sent for analysis. All mammary glands referred to were evaluated histologically and classified according to Cassali et al. (2020).

**Figure 1:**
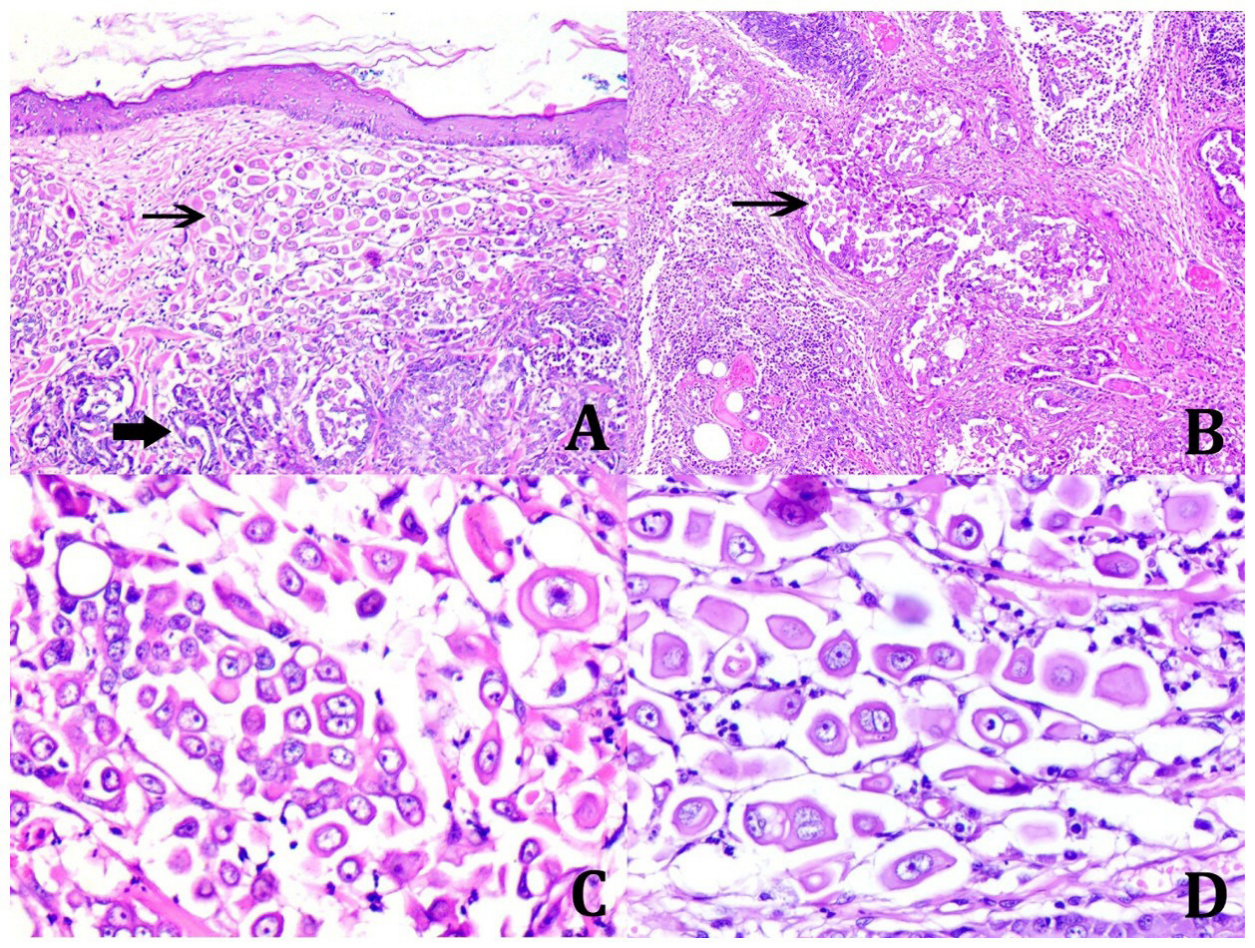
Histological features of lobular pleomorphic carcinoma in a cat. A. In the mammary gland and invading the dermis, two neoplastic proliferations were observed: tubulopapillary carcinoma (thick arrow) and lobular pleomorphic carcinoma (thin arrow), with invasive areas in the superficial dermis. B. The lobular pleomorphic carcinoma also exhibited in situ areas (arrow), primarily within the mammary gland, where cells were loosely arranged inside the mammary ducts. C and D. In the invasive areas, cells were arranged in an Indian file pattern or dispersed within the stroma, exhibiting marked pleomorphism, with large nuclei, prominent nucleoli, frequent karyomegaly, bizarre nuclei, and multinucleation.

Histologically, two neoplastic proliferations with distinct characteristics were observed in the caudal abdominal mammary gland (Figures 1A and 1B). The first was a multinodular epithelial neoplastic proliferation, composed of epithelial cells arranged in tubular and papillary patterns, sometimes forming cribriform areas (Figure 1A, thick arrow). This neoplasm expanded the mammary gland and infiltrated the subcutaneous tissue. It consisted of cells with scant eosinophilic cytoplasm, oval nuclei, coarse chromatin, and moderately evident nucleoli, showing marked pleomorphism and a high mitotic count (tubulopapillary carcinoma, grade III). The second was a neoplastic proliferation formed by isolated cells arranged in an Indian file pattern or dispersed within the stroma (Figure 1A, thin arrow), interspersed with dense connective tissue (invasive areas, Figure 1A, thin arrow), as well as loosely arranged inside ducts (in situ areas, Figure 1B, thin arrow). This proliferation expanded the mammary gland, infiltrated the first tumor, and formed satellite nodules that extended to the dermis and subcutaneous tissue. The cells exhibited a moderate nucleus-to-cytoplasm ratio, slightly eosinophilic and wide cytoplasm, rounded to oval nuclei, coarse chromatin, and prominent nucleoli. Marked anisocytosis and anisokaryosis, frequent karyomegaly, multinucleation, cells with bizarre nuclei (Figures 1C and 1D, and 24 mitotic figures per 2.37 mm^2^ were observed (pleomorphic lobular carcinoma, grade III). Extensive areas of necrosis and vascular invasion by neoplastic cells were noted in the dermis and subcutaneous tissue, with histological features like those of pleomorphic lobular carcinoma. The overlying epidermis was extensively ulcerated, and there was an intense histiocytic and neutrophilic inflammatory infiltrate, associated with lymphocytes. The combination of these histopathological characteristics led to the diagnosis of grade III tubulopapillary carcinoma and grade III pleomorphic lobular carcinoma.

In the cranial abdominal mammary gland, a neoplastic proliferation was observed, composed of epithelial cells arranged in tubular and papillary patterns, with both in situ and invasive carcinomatous areas, interspersed with dense connective tissue. This proliferation expanded the mammary gland and formed satellite nodules that infiltrated the dermis, exhibiting histological characteristics like those observed in the caudal abdominal mammary gland. The combination of these histopathological features led to the diagnosis of grade II tubulopapillary carcinoma.

In the lymph node, the normal architecture was lost in some areas due to neoplastic proliferation exhibiting tubulopapillary and cribriform areas, as well as nests and rows of neoplastic cells characteristic of the pleomorphic lobular pattern replacing the parenchyma. These features were consistent with those observed in the caudal abdominal mammary gland, and the lesion measured 0.6 cm in its largest axis, consistent with a macrometastasis of carcinoma, as defined by Araújo et al. (2015). Foci of necrosis were also observed in the metastatic areas. Additionally, areas of epithelial proliferation were noted extending beyond the nodal capsule (extracapsular extension). Clinically, due to the presence of metastasis in the inguinal lymph node, the patient was classified as stage IV (Cassali et al., 2020).

Fragments of the tumor diagnosed in the caudal abdominal mammary gland were sent for immunohistochemical evaluation. Multiple 3-µm-thick sections were placed on gelatin slides and submitted to the immunohistochemical technique. Table 1 shows the antibodies used, clone, dilution and antigen retrieval method, with DAB (DAB substrate system, Dakocytomation) being used as the chromogen for three minutes. Positive controls were used, and negative controls were obtained by replacing the primary antibody with normal serum.

**Table 1:** Antibodies and immunohistochemical protocol used.

**Target Antigen**	**Clone**	**Dilution**	**Antigen Retrieval Method**
RE	Monoclonal Rabbit Anti-Human (EP1)	Ready to use	Pressurized Heat (125ºC/2min) whit citrate buffer pH 6.0
RP	Monoclonal Mouse Anti-Human (HPRA2)	1:50	Pressurized Heat (125ºC/2in) whit citrate buffer pH 6.0
COX-2	Monoclonal Rabbit Anti-Human (SP21)	1:50	Pressurized Heat (125ºC/2min) whit citrate buffer pH 6.0
HER-2	Polyclonal Rabbit Anti-Human	1:200	Pressurized Heat (125ºC/2min) whit citrate buffer pH 6.0
E-CAD	Monoclonal Mouse Anti-Human (EP700Y)	1:50	Pressurized Heat (125ºC/2min) whit citrate buffer pH 6.0
P63	Monoclonal Mouse Anti-Human (DAK -p63)	1:100	Water-bath Heating (95°C/20min) with citrate buffer pH 6.0
Ki67	Monoclonal Mouse Anti-Human (MIB-1)	1:50	Pressurized Heat (125°C/2min) with citrate buffer pH 6.0

**Figure 2:**
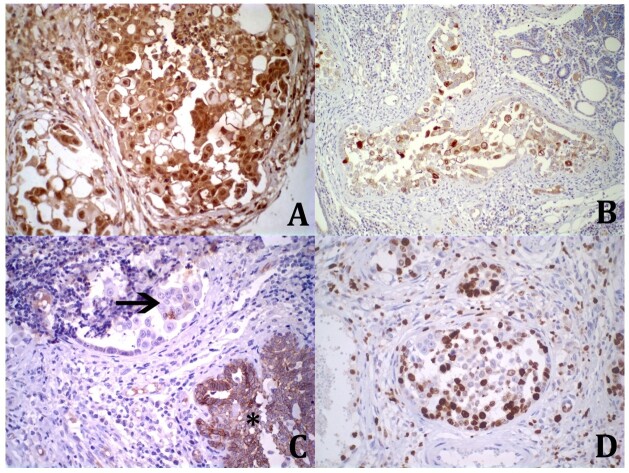
Immunohistochemical features of lobular pleomorphic carcinoma in a cat. Cells were strongly positive for the progesterone receptor (A) and scored 3 for cyclooxygenase 2 (B). For E-cadherin, the neoplastic cells of the lobular pleomorphic carcinoma were negative (arrow), while the cells of the tubulopapillary carcinoma were positive (asterisk). The Ki67 marker showed positive nuclear staining in approximately 90% of the neoplastic cells (D).

The neoplasm was positive for progesterone receptor (PR) in 75% of the neoplastic cells (Figure 2A) and for the cyclooxygenase 2 (COX-2) marker (Figure 2B), with a score of 3 (strong cytoplasmic immunostaining in <10% of cells, Lavalle et al., 2009). It was negative for estrogen receptor (ER), HER-2, E-cadherin (Figure 2C, arrow), and P63. Additionally, the Ki67 marker showed positive nuclear staining in approximately 90% of the neoplastic cells (Figure 2D).

Approximately 15 days after the surgical procedure, the patient received clinical care from an oncologist, who identified an enlarged inguinal lymph node in the left mammary chain, multiple nodules in different glands of the chain, and an enlarged right axillary lymph node. Imaging tests were ordered to monitor for possible metastases. These tests, conducted approximately 25 days after the initial imaging (before the first surgery), showed no signs of intrathoracic neoplasia. At the owner's request, surgery was postponed for a month, and approximately 43 days after the first surgery, the cat returned for care and additional tests for a subsequent mastectomy of the left mammary chain. The nodules in the mammary chain had increased in size, and the patient presented with hyporexia and respiratory distress; therefore, the cat was hospitalized. A chest X-ray revealed the presence of free fluid in the thoracic cavity, and three drainages were performed on consecutive days, resulting in 80 mL in the first, 70 mL in the second, and 100 mL in the third drainage. The fluid was sent for cytological evaluation, which indicated neoplastic effusion of probable epithelial origin. Five days after hospitalization and 48 days following the first surgical procedure, the patient developed complete paralysis of the pelvic limbs and worsening respiratory signs. The patient was euthanized due to poor prognosis. An autopsy examination was not performed.

## Discussion

Based on the histopathological characteristics, the tumor in this case was diagnosed as tubulopapillary carcinoma and pleomorphic lobular carcinoma, both with histological grade III, exhibiting vascular invasion of the dermis and subcutaneous tissue, as well as macrometastasis with extracapsular extension in the regional lymph node, characterizing clinical stage IV at the time of diagnosis. Pleomorphic lobular carcinoma is characterized by diffuse infiltration of cells with marked pleomorphism within the stroma, either isolated and dispersed or forming cord-like structures (Cassali et al., 2002; Jacobs et al., 2012; Lokuhetty et al., 2019). The cellular features of karyomegaly, multinucleation, and the presence of cells with bizarre nuclei, as observed in the case described here, are important diagnostic aspects, in addition to the characteristic histologic pattern (Al-Baimani et al., 2015; Jacobs et al., 2012; Lokuhetty et al., 2019).

Mammary carcinomas in cats are characterized by clinical and histopathological aggressiveness, with frequent systemic progression (Cassali & Nakagaki, 2023; Souza et al., 2024), as observed in the cat in this case. The metastatic rate reaches 90%, most commonly affecting the lungs, lymph nodes, liver, and pleura (Cassali & Nakagaki, 2023; Zappulli et al., 2015). Lymph node or systemic involvement clinically characterizes stage IV (Cassali et al., 2020). At the time of mastectomy, due to the involvement of the inguinal lymph node, the cat in this report was classified as stage IV. However, it did not present systemic clinical signs or evidence of metastasis on the imaging exams performed, which could suggest longer survival, as reported by Petrucci et al. (2021). According to this study, the survival of cats with stage IV and systemic clinical signs at the time of diagnosis was on average 14 days, while those without systemic signs, despite the staging, had an average survival of 128 days. Cats that developed pleural effusion had a survival time of 16 days (Petrucci et al., 2021). Approximately 40 days after the surgical procedure, the patient developed respiratory signs, with pleural effusion diagnosed as neoplastic through cytological exams. This was followed by a significant worsening of the clinical condition and the development of neurological symptoms, consistent with findings in the literature (Petrucci et al., 2021). Due to the poor prognosis, the patient was euthanized.

In humans, pleomorphic lobular carcinomas are recognized as a variant of invasive lobular carcinoma, with an unfavorable prognosis and shorter survival compared to other histological types of breast tumors (Haque et al., 2019; Sağdıç et al., 2024; Zhang et al., 2022). This important characteristic confers malignancy by promoting marked invasion of the breast and adjacent tissues, including lymphatic vessels, which often leads to the development of distant metastases (Sağdıç et al., 2024). In the case described here, both characteristics were observed: vascular invasion and metastasis in regional lymph nodes. Although imaging tests at the time of mastectomy did not show evidence of systemic involvement, as the case progressed, the patient developed respiratory signs due to neoplastic pleural effusion, neurological signs, an increase in nodules in the contralateral chain, and involvement of axillary lymph nodes. These findings highlight the aggressive behavior of the neoplasm in cats, like that observed in humans (Haque et al., 2019; Sağdıç et al., 2024; Zhang et al., 2022).

Several studies have demonstrated that pleomorphic lobular carcinomas have higher Ki67 values and higher histological grades compared to other types of invasive carcinomas of the female mammary gland (Jacobs et al., 2012; Jung et al., 2013; Monhollen et al., 2012; Sağdıç et al., 2024; Zhang et al., 2022). Similarly, the tumor diagnosed in this case was grade III according to the World Health Organization grading system, with clinical stage IV at the time of diagnosis and a high Ki67 percentage, showing a proliferative index of 90%. The development of neurological and respiratory signs as the clinical picture progressed indicated progression to stage V.

The hormonal immunohistochemical profile of the cat in this report showed negativity for ER and HER-2, and positivity for PR. In women, previous studies indicate that pleomorphic lobular carcinoma is more likely to be negative for hormone receptors compared to invasive lobular carcinoma (Haque et al., 2019; Jacobs et al., 2012; Sağdıç et al., 2024; Zhang et al., 2022). Furthermore, as observed in women (Jacobs et al., 2012; Haque et al., 2019), there was a loss of E-cadherin staining. However, for felines, there are still no studies in the literature that have proven a correlation between survival and hormonal expression (Cassali et al., 2020; Hughes & Dobson, 2012; Millanta et al., 2006).

In cats, a case with histological characteristics like those observed in this case has been described in the literature, diagnosed as anaplastic carcinoma according to the histological classification adopted by the authors (Soares et al., 2021). In that case, the presence of highly pleomorphic epithelial cells arranged in small groups with bizarre nuclei led to the histopathological diagnosis of anaplastic carcinoma. Due to similar histological features, it is possible that this tumor is a pleomorphic lobular carcinoma, according to the classification proposed by Cassali et al. (2020) and the WHO (Lokuhetty et al., 2019). Like the cat in this report, there was involvement of regional lymph nodes and invasion of the dermis by neoplastic cells. The immunohistochemical findings were also similar, with negativity for ER and positivity for pancytokeratin and PR (Soares et al., 2021). However, the tumor in this report was negative for HER-2.

## Conclusion

Pleomorphic lobular carcinomas are highly aggressive tumors with distinctive histological characteristics that are key to their diagnosis. Although uncommon in felines, this tumor should be considered as a potential differential diagnosis, primarily due to its high metastatic potential and aggressive behavior.
